# Inhibition of HIV-1 gene expression by Sam68ΔC: multiple targets but a common mechanism?

**DOI:** 10.1186/1742-4690-6-22

**Published:** 2009-03-02

**Authors:** Alan Cochrane

**Affiliations:** 1 Department of Molecular Genetics, University of Toronto, Toronto, Ontario, Canada

## Abstract

Two recent publications have explored the mechanisms by which a mutant of the host protein Sam68 blocks HIV-1 structural protein synthesis and expands its activity to encompass Nef. Although the two studies propose different mechanisms for the responses observed, it is possible that a common activity is responsible. Understanding how this Sam68 mutant discriminates among the multiple viral mRNAs promises to reveal unique properties of HIV-1 RNA metabolism.

## Commentary

One of the principles underlying the use of any compound or factor as a therapeutic agent is its capacity to selectively affect the target with little or no off-target effects. With this concept in mind, recent reports regarding the ability of a variant of the host factor Sam68 to selectively regulate the expression of several key components of HIV-1 take on particular interest. HIV-1 replication is critically dependent on the expression of its structural proteins, Gag, Gagpol and Env [1]. As a result, any factor able to inhibit expression of these proteins would force the virus into a state akin to latency. In addition, HIV-1 Nef has been implicated as a major player in the pathogenesis of this virus [2,3], expression of Nef alone in transgenic mice reproducing many aspects of the pathology seen by the intact virus in humans [4]. The recent reports that a mutant of Sam68, Sam68ΔC (lacking the C-terminal nuclear localization signal), is able to interfere at both the level of HIV structural protein and Nef synthesis makes it of particular interest [5,6].

**Figure 1:**
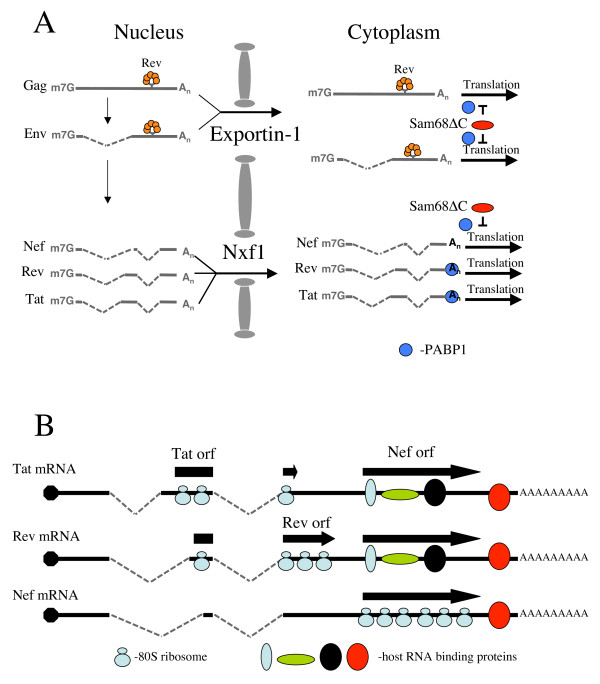
**Understanding regulation of HIV-1 gene expression by Sam68ΔC**. (A) Following transcription, HIV-1 RNA undergoes alternative splicing to generate over 40 mRNAs that correspond to unspliced (encoding Gag and Gagpol), singly spliced (to produce Vif, Vpr, Vpu and Env) or multiply spliced (for generating Tat, Rev and Nef) mRNAs. Unspliced and singly spliced viral RNAs are exported to the cytoplasm via exportin-1, which is mediated by Rev, while the multiply spliced RNAs exit using Nxf1. Once within the cytoplasm, Sam68ΔC interacts with the unspliced, singly spliced and *nef *mRNAs to block their translation by preventing the binding of PABP1 (shown as a small blue circle). In contrast, PABP1 binds to *tat *and *rev *mRNAs, and translation is unaffected. (B) A model for the discrimination between *tat, rev *and *nef *mRNAs. The process of splicing used to generate the mRNAs encoding Tat, Rev and Nef results in slight variations in 5' sequence, but all the mRNAs encompass the *nef *reading frame (individual reading frames are illustrated by block arrows). However, translation of the individual reading frames could result in variations in the composition/structure of the mRNA within the common sequence (as represented by the coloured ovals). Such differences in composition/structure of the viral mRNP could serve as means by which Sam68ΔC selectively regulates their expression.

Initial experiments [7] identified Sam68ΔC as a dominant inhibitor of HIV-1 replication. While subsequent work determined that inhibition was dependent upon the cytoplasmic localization of Sam68ΔC and associated with formation of cytoplasmic granules around the outside of the nuclear envelope [8], the underlying mechanism remained unclear. However, the recent work of Marsh *et al. *[6] provided some detail as to the mechanism. Using various expression vectors, they showed that Sam68ΔC selectively inhibited mRNA expressing Gag exported via the exportin-1 pathway, with little to no effect on the same Gag coding sequence delivered to the cytoplasm by Nxf1. Co-expression of Sam68ΔC and an unspliced Env expressor resulted in translation inhibition of the latter and disruption of the cytoplasmic bundles failed to restore expression of the encoded protein. Rather, despite a normal polyA tail, inhibition by Sam68ΔC was attributed to a block in translation of the affected RNA due to reduced binding of PABP-1 (Fig. 1A). The ability of Sam68ΔC to selectively affect only those RNAs exported in a Rev- and exportin-1-dependent fashion suggested that it recognizes some features unique to the mRNPs exported by this pathway. In parallel work by Henao-Mejia *et al. *[5] and consistent with Marsh *et al*., it was shown that constructs functionally similar to Sam68ΔC had the capacity to repress Rev-dependent protein expression. Surprisingly, inhibition of Rev-independent Nef synthesis was also observed with little or no alteration in Tat or Rev levels. Given that these three proteins are expressed from multiply spliced HIV-1 RNAs that all use the Nxf1 export pathway (Fig. 1A), selective repression of Nef expression may require a different mechanism than that outlined by Marsh *et al*. Inhibition of Nef expression was reported to be associated with the accumulation of *nef *mRNA in cytoplasmic granules that co-stained with markers of stress granules (SGs); these observations led Henao-Mejia *et al. *to suggest that reduced Nef synthesis was due to sequestration in these bodies. At present, it is unclear whether the granules characterized by Henao-Mejia *et al. *are similar or distinct from those formed by Sam68ΔC and incompletely spliced HIV-1 RNAs and whether Sam68ΔC inhibition of Nef synthesis is dependent upon their integrity. The two studies suggest that, while the route different RNAs take to repressive sites can differ (the Exportin1 pathway for Rev-dependent RNAs versus the Nxf1 pathway for *nef *mRNA), a similar mechanism may underlie repression of HIV-1 structural protein and *nef *mRNAs by Sam68ΔC. However, whether the mechanism is simple sequestration in SGs or something more complex remains to be determined. This is based on the observation of Marsh *et al*. that RRE-containing RNAs are still repressed upon dispersal of Sam68ΔC granules, although dispersion into functional "nano" granules cannot be dismissed and should be investigated. In addition, ongoing studies (Marsh and Cochrane, unpublished) showing that Sam68ΔC-induced granules contain mRNAs whose expression is not repressed suggest that sequestration to such granules alone is insufficient to explain translational repression. Consequently, additional experiments are needed to assess whether common or distinct mechanisms underlie repression of HIV-1 structural protein and *nef *mRNAs by Sam68ΔC.

The suggestion that Sam68ΔC can discriminate *nef *mRNA from that of *tat *and *rev *is of particular interest given that these RNAs not only share a common export pathway but are almost identical except for differences in their 5' untranslated regions (Fig. 1B). The determination by Henao-Mejia *et al. *that sensitivity to Sam68ΔC is due to sequences in the 3'UTR of *nef *mRNA that are also present in *tat/rev *mRNAs raises questions about how repression is restricted to *nef *mRNAs. One hypothesis is based on the position of the different reading frames and the influence of translation on 3' UTR structure/RNP composition. Both *tat *and *rev *mRNAs contain reading frames encoding the respective proteins (Tat or Rev) and that of Nef, while *nef *mRNA has only one reading frame (Fig. 1B). Since translation requires the unfolding of RNA secondary structure as well as disruption of protein-RNA interactions, it is possible that the sequence spanning the Nef reading frame within *tat *and *rev *mRNAs could have very different secondary structure and/or RNP composition than *nef *mRNA. Consequently, repression specificity could be achieved by Sam68ΔC binding to RNPs containing alternative structure/composition in the region common to the three mRNAs. Such a hypothesis is readily testable and will provide important insights into the determinants that specify susceptibility to regulation by Sam68ΔC. Defining the mechanism by which Sam68ΔC selectively inhibits the expression of several key HIV-1 mRNAs will provide important insights into their regulation and potentially lead to new approaches to controlling the pathogenesis of this virus.
